# Switching off DNA repair—how colorectal cancer evades targeted therapies through adaptive mutability

**DOI:** 10.1038/s41392-020-0120-3

**Published:** 2020-02-21

**Authors:** Jörg Fahrer

**Affiliations:** Division of Food Chemistry and Toxicology, Department of Chemistry, Technical University of Kaiserslautern, 67663 Kaiserslautern, Germany

**Keywords:** Gastrointestinal cancer, Preclinical research, Cancer genetics, Gastrointestinal cancer, Preclinical research

**Fig. 1:**
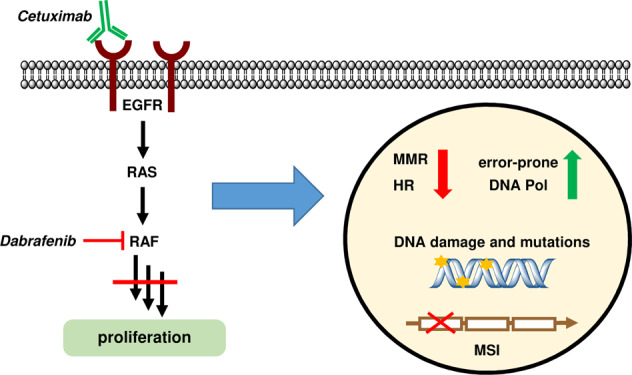
Colorectal cancer cells downregulate DNA repair pathways (MMR and HR) and upregulate error-prone DNA polymerases in response to targeted therapies, thereby promoting adaptive mutability and microsatellite instability (MSI)

**A very recent study by Russo et al. published in*Science*demonstrates that colorectal cancer (CRC) cells adapt to targeted therapies by downregulating DNA repair at the expense of an increased mutation frequency and microsatellite instability(MSI).** ^1^**The authors intriguingly showed that CRC cells are capable of activating stress-induced mutagenesis similar to unicellular organisms in a transient and controlled manner, allowing them to survive under targeted therapies (Fig.** 1**).**

CRC is a very common cancer type that is tightly associated with multiple genetic, lifestyle, and nutritional risk factors.^2,3^ In the course of the disease, ~50% of CRC patients develop metastases with poor prognosis. These late-stage patients receive chemotherapy along with targeted therapies, which are directed against the epidermal growth factor receptor (EGFR) and vascular endothelial growth factor (VEGF) as the main targets in metastatic CRC.^4^ Although these targeted therapies show clinical efficacy and prolong survival in metastatic patients, resistance almost inevitably occurs, resulting in disease progression and death.^5^

The Alberto Bardelli group hypothesized that human CRC cells can increase their mutation rate in response to targeted therapies, thereby gaining a survival benefit.^1^ This phenomenon is well known to occur in bacteria under stress conditions such as antibiotic treatment, and is attributable to a reduction in DNA mismatch repair (MMR) capacity and an upregulation of error-prone DNA polymerases.^6,7^ Russo et al. addressed this issue in microsatellite-stable (MSS) CRC cell lines and patient-derived xenograft (PDX) models, which were treated with the monoclonal anti-EGFR antibody cetuximab (CTX) or a combination of CTX and the BRAF inhibitor dabrafenib. The researchers further extended their study to a clinical setting with two patients receiving chemotherapy together with anti-EGFR therapy.

Using RNA-seq and Q-PCR, the authors first showed a downregulation of genes involved in MMR (e.g., MLH1 and MSH6), and in homologous recombination (HR) repair (e.g., BRCA1/2 and Rad51) in drug-tolerant persister cells after CTX or CTX plus dabrafenib treatment. The suppression of MMR and HR was also detected at the protein level, translating into reduced repair capacities as revealed by plasmid-based repair assays. Concomitantly, error-prone DNA polymerases (e.g., Pol *iota*, Pol *kappa*) were upregulated. Intriguingly, the alterations induced in persister cells by targeted therapies closely resemble the phenotype observed in bacteria under stress conditions that engage adaptive mutability. Consistent with these findings, the suppression of both MMR and HR was maintained in persister cells, but their expression was restored to normal levels after removal of the targeted therapy, highlighting the transient nature of this phenotype. Importantly, the authors also confirmed the downregulation of MMR proteins in different PDX models, and in two patients receiving anti-EGFR therapy.

The compromised DNA repair in persister cells resulted in elevated DNA damage levels, as revealed by immunofluorescence staining of γ-H2AX and 53BP1. The authors provided further evidence that CTX treatment causes the formation of reactive oxygen species (ROS) as a possible source of DNA damage. Whether and how CTX-induced ROS formation is causally linked to genetic instability in persister cells deserves further attention. To elucidate the mechanistic basis and to delineate this active stress response pathway, the authors used different stimuli and analyzed their effects on the DNA repair machinery of CRC cells. Direct DNA damage by oxaliplatin or thymidine-mediated cell cycle stress rather stimulated MMR and HR expression, whereas these parameters were unaffected by nutrient deprivation. Furthermore, the authors observed downregulation of the mammalian target of rapamycin (mTOR) and mTOR-dependent signaling by the targeted therapies, with comparable kinetics to those observed for MMR and HR. To identify a causal link between mTOR suppression and the downregulation of DNA repair genes, the authors performed a transient knockdown of mTOR; however, no effects on MMR or HR components were detected. Genetic abrogation of *EGFR, KRAS*, or *BRAF* in CRC cells mimicked the effects observed by pharmacological inhibition, leading to a downregulation of MMR and HR, elevated ROS and DNA damage levels, as well as mTOR suppression.

Next, the authors wanted to know whether the compromised DNA repair and elevated levels of error-prone polymerases influence the de novo mutagenesis in persister cells. Using a reporter assay based on the so-called CA-NanoLuc vector, Russo and colleagues were indeed able to identify random mutations in persister cells exposed to EGFR and BRAF inhibition. This was further analyzed by whole-exome sequencing of microsatellite regions, which are known for their instability in the absence of MMR. Their experiments showed significant alterations in the length of the microsatellite regions in both persister and drug-resistant cells, which were also found in a PDX model treated with CTX until resistance developed.

Altogether, the study by Russo and colleagues elegantly illustrates how CRC cells can evade targeted therapies by switching off DNA repair pathways, and thereby increasing mutation rates in a transient and controlled manner. Their findings have clinical implications, and might lead to novel treatment options for patients with late-stage disease. The observed downregulation of HR may sensitize persister CRC cells to treatment with poly(ADP-ribose) polymerase-1 (PARP-1) inhibitors such as olaparib, thus enabling a synthetic lethality approach. PARP-1 and its product PAR were found to be overexpressed in human colorectal cancer biopsies, and PARP-1 was shown to drive colorectal tumor progression in vivo.^8^ Very recently, a subset of colorectal cancers with deficiency in HR was identified, which displayed increased susceptibility to PARP inhibition, and at the same time, cross-sensitivity to oxaliplatin treatment.^9^ Furthermore, the MMR deficiency and the concomitant mutability induced by EGFR/BRAF inhibitor therapy likely promote neoantigen formation and immune cell activation, thus suggesting a combination with immunotherapy. The immune checkpoint inhibitors pembrolizumab and nivolumab have been recently approved by the FDA for the treatment of refractory CRC with MMR deficiency and high levels of MSI.^10^ Future clinical studies considering these aspects are warranted, and may help to improve the clinical efficacy of targeted therapies.
